# Evaluation of pituitary function and metabolic parameters in patients with traumatic maxillofacial fractures

**DOI:** 10.1007/s40618-024-02349-8

**Published:** 2024-03-19

**Authors:** O. O. Daloglu, M. C. Unal, C. A. Kemaloglu, O. F. Bolatturk, I. Ozyazgan, F. Tanriverdi, A. Coruh, F. Kelestimur

**Affiliations:** 1 Department of Endocrinology and Metabolism, Bursa Yuksek Ihtisas Training and Research Hospital, University of Health Sciences, Bursa, Turkey; 2 Department of Endocrinology and Metabolism, Dokuz Eylul University Faculty of Medicine, Izmir, Turkey; 3 Department of Plastic Surgery and Reconstruction, Erciyes University Faculty of Medicine, Kayseri, Turkey; 4 Department of Neurology, Erciyes University Faculty of Medicine, Kayseri, Turkey; 5 Endocrinology Clinic, Memorial Kayseri Hospital, Kayseri, Turkey; 6 Department of Endocrinology, Faculty of Medicine, Yeditepe University, Istanbul, Turkey

**Keywords:** Maxillofacial fracture, Traumatic brain injury, Pituitary, Hypopituitarism, Growth hormone deficiency

## Abstract

**Purpose:**

This study was designed to assess the pituitary functions of patients with traumatic maxillofacial fractures and compare the results with healthy controls.

**Methods:**

Thirty patients (mean age, 38.14 ± 14.15 years; twenty-six male, four female) with a traumatic maxillofacial fracture at least 12 months ago (mean 27.5 ± 6.5 months) and thirty healthy controls (mean age, 42.77 ± 11.36 years; twenty-five male, five female) were included. None of the patients were unconscious following head trauma, and none required hospitalization in intensive care. Basal pituitary hormone levels of the patients were evaluated. All patients and controls had a glucagon stimulation test and an ACTH stimulation test to evaluate the hypothalamic–pituitary–adrenal axis and the GH–IGF-1 axis.

**Results:**

Five of thirty patients (16.6%) had isolated growth hormone (GH) deficiency based on a glucagon stimulation test (GST). The mean peak GH level after GST in patients with hypopituitarism (0.54 ng/ml) was significantly lower than those without hypopituitarism (7.01 ng/ml) and healthy controls (11.70 ng/ml) (*P* < 0.001). No anterior pituitary hormone deficiency was found in the patients, except for GH.

**Conclusion:**

Our study is the first to evaluate the presence of hypopituitarism in patients with traumatic maxillofacial fractures. Preliminary findings suggest that hypopituitarism and GH deficiency pose significant risks to these patients, particularly during the chronic phase of their trauma. However, these findings need to be validated in larger scale prospective studies with more patients.

## Introduction

Traumatic brain injury (TBI) is a common health issue that is one of the leading causes of death and disability among young adults. Maxillofacial trauma is one of the most common forms of head trauma. There is an increased prevalence of neuroendocrine dysfunction in patients with TBI [1–4]. TBI can cause partial or complete hypopituitarism, and 25–50% of patients have been reported to have pituitary dysfunction [1, 3, 5]. Gonadotropins and growth hormone (GH) are the most commonly deficient pituitary hormones, and they appear to be easily affected even after mild TBI [3, 5]. Although several case reports of spontaneous recovery have been published [6–8], posttraumatic hypopituitarism is generally accepted to be permanent.

Recent research has shown that amateur boxing and kickboxing-related head trauma can result in pituitary hormone deficiencies, particularly GH deficiency (GHD) [9–12]. When compared to other causes of moderate traumatic brain injury (MTBI), such as a car accident or a fall, the intensity of the head trauma is lower, and the pattern of the head trauma is slightly different, indicating chronic repetitive MTBI.

The maxillofacial region plays a key role in ensuring normal feeding, chewing, breathing, and craniocerebral preservation. If maxillofacial fractures are not treated appropriately and promptly, they can have a substantial damaging influence on patients.

In this study, we aimed to analyze head trauma cases with maxillofacial trauma and evaluate their pituitary functions. Long-term hormonal changes, lipid profiles, and anthropometric measurements following head trauma are also investigated, as is their relationship with pituitary insufficiency.

## Materials and methods

### Subjects

**Fig. 1:**
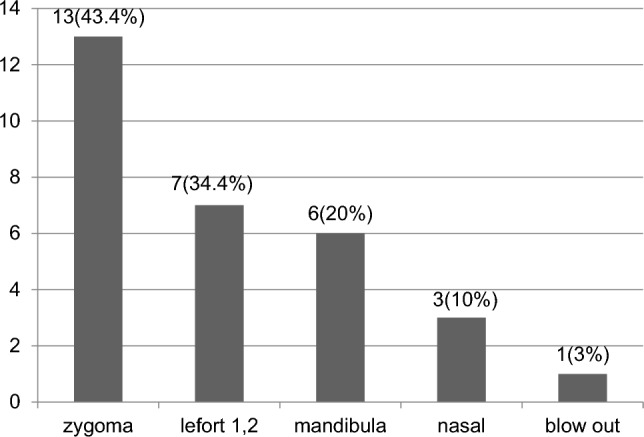
Type of fracture in patients with maxillofacial trauma

The study included 30 patients with maxillofacial fractures (mean age, 38.14 ± 14.15 years, min–max, 18–65 years; 26 male, 4 female) and sex-matched 30 healthy controls (mean age, 42.77 ± 11.36 years, min–max, 20–63 years; 25 male, 5 female). In both groups, subjects within 18–65 years were included. In the control group, none had acute or chronic illnesses affecting the hypothalamic–pituitary axis including infection, malignancy, radiotherapy, endocrinological diseases or a history of drug use such as glucocorticoids. Patients included in the study were retrospectively screened. These patients had a history of maxillofacial fracture at least a year before (mean: 27.5 ± 6.5 months) and were monitored by the Erciyes University Medical School Plastic Surgery and Reconstruction Department. Patients did not have any chronic diseases, such as diabetes mellitus or chronic kidney disease, nor did they have a history of medication use, including corticosteroids. The following were the causes of head trauma; 13 patients had zygomatic fractures, 7 patients had Le fort 1–2, 6 patients had mandibular fractures, 3 patients had nasal fractures, and 1 patient had a blow-out fracture (Fig. 1). The level of consciousness of the patients was evaluated with Glasgow Coma Scale (GCS). A score of 13–15 indicates mild TBI, 9–12 indicates moderate TBI, and ≤ 8 indicates severe TBI. Patients with mild head trauma were included in the study group. All of the patients included in the study had GCS 15 during and after the trauma, according to hospital records and patient history. The ethics committee and the institutional review board of Erciyes University Medical School approved this study, and informed consent was obtained from each patient and control subject (Project number: TTU-2015-5747).

### Assessment of patient characteristics and biochemical parameters

Pituitary dysfunction was defined by basal hormone levels and/or hormonal response to dynamic tests. Internationally accepted criteria were used to diagnose pituitary hormone deficiencies, as detailed below. Both patients and controls were outpatients. Laboratory tests were performed after a thorough physical examination that included arterial blood pressure and heart rate. Lipid profile (total cholesterol, high-density lipoprotein cholesterol, triglycerides), fasting plasma glucose level, renal function tests (blood urea nitrogen, creatinine, uric acid, and electrolytes), liver function tests (alanine aminotransferase, aspartate aminotransferase, total alkaline phosphatase, total protein, albumin levels), and complete blood count were measured at the Department of Clinical Biochemistry, Erciyes University. Insulin resistance was assessed by calculating the homeostatic model assessment score (HOMA) using the formula: (fasting glucose [mg/dl] × fasting insulin [mU/l]/405).

### Assessment of pituitary function

#### Basal hormone levels

Basal hormonal parameters were measured, including free T3, free T4, thyroid-stimulating hormone (TSH), prolactin, follicle-stimulating hormone (FSH), luteinizing hormone (LH), total testosterone in males, estradiol in women, GH and insulin-like growth factor 1 (IGF-I) levels.

In men, hypogonadotropic hypogonadism was defined as a total testosterone value of < 134 ng/dl in the presence of normal or low values of gonadotropins (reference range; FSH 0.95–11.95, LH 0.57–12.07). Hypogonadotropic hypogonadism was defined in premenopausal women as a serum estradiol level ≤ 11 pg/ml combined with an abnormally low or normal serum gonadotropin concentration (LH—follicular phase: 1.80–11.78 mIU/ml, ovulation phase: 7.79–89.08 mIU/ml, luteal phase: 0.56–14.00 mIU/ml; FSH—follicular phase: 3.03–8.8 mIU/ml, ovulation phase: 2.55–16.69 mIU/ml, luteal phase: 1.38–5.43 mIU/ml) [13, 14]. In women, menstrual history was obtained, and tests were performed on days 3–4 of the follicular phase. TSH deficiency was defined as a low serum-free T4 level (< 7.7 pg/ml) without an increase in serum TSH [13, 14].

### Assessment of somatotropic and corticotropic function and definition of abnormalities

A low-dose ACTH stimulation test and glucagon stimulation test (GST) were used to assess the GH–IGF-I and ACTH–cortisol axis in patients and controls.

All patients and controls received a glucagon test (1 mg intramuscular glucagon; Novo Nordisk; with blood sampling for cortisol and GH at baseline, 90, 120, 150, 180, 210, and 240 min) to establish normal cortisol and GH response to glucagon stimulation, the cutoff value was estimated from the cortisol and GH responses of healthy controls. The GH response to glucagon was greater than 1.18 µg/l in all 30 healthy subjects (median 11.7 ± 11.45 μg/l; range 1.18–36.79 µg/l). Therefore, we took a cutoff of 1.18 µg/l as a normal GH response after glucagon administration [4, 11]. Cortisol levels in all 30 healthy subjects exceeded 9.1 µg/dl (median 21.17 ± 6.58 μg/dl; range 9.1–52.32 µg/dl). Therefore, we took 9.1 µg/l as a cutoff value of normal cortisol response [15]. Furthermore, a low-dose ACTH stimulation test with 1 µg tetracosactide intravenous (Synacthten, Novartis Pharma, Lion, France) was performed on all patients and controls, as previously described, and serum samples were obtained for cortisol measurement basally and at 30, 60, 90, and 120 min. ACTH deficiency was defined as a peak cortisol level of less than 12.5 µg/dl [15].

### Analytic methods of hormonal parameters

The intra-assay and inter-assay coefficients of variation (CV) for serum GH were 1.5 and 14%, respectively. The minimum detection limit was 0.01 µg/l, and GH standards were calibrated according to the World Health Organization reference standard 88/624. The intra-assay and inter-assay for IGF-I were 3.4 and 8.2%, respectively, after formic acid–ethanol extraction (DSL). All the other serum hormones were measured using radioimmunoassay, IRMA, or chemiluminescent methods with the following commercial kits; cortisol (DSL; intra-assay and inter-assay CV: 8·4% and 9·1%), TSH-IRMA (Izotop; 7·3% and 3·8%), PRL (Advia Centaur System Chemiluminescent Technology, Bayer, Germany; 3·3% and 4·7%) sT3 (Izotop, Budapest, Hungary; 22% and 81%), sT4 (Izotop; 34% and 55%, FSH (Advia Centaur System Chemiluminescent Technology; 2·9% and 2·7%), LH (Advia Centaur System Chemiluminescent Technology; 2·3% and 1·5%), total testosterone (Biosource, Nivelles, Belgium; 4·6% and 6·2%), and estradiol ( 5·5% and 5·2%).

### Statistical methods

Statistical analysis was performed using the SPSS 20.0 program. For normality, all data were subjected to the Kolmogorov–Smirnov test and Shapiro–Wilk test. Normally distributed values were presented as mean ± standard deviation (SD). Non-normally distributed values were presented as median (interquartile range). The ANOVA test was used to compare the differences between GH-deficient, GH-sufficient, and control groups for normally distributed values. The Kruskal–Wallis test and Mann–Whitney *U* test were used to compare the differences between subjects for the non-normally distributed values. The correlation between the variables was analyzed using Pearson’s correlation analysis. Linear regression analysis was used to determine the effect of independent variables on hormonal parameters. The significance level was determined as *P* value of < 0.05.

## Results

The patients included in the study suffered a head injury between November 2011 and September 2014. At least 1 year has passed since head trauma on the date they were taken to study (mean: 27.5 ± 6.5 months). Patients in the study were divided into GH-deficient and GH-sufficient groups based on stimulation tests. The demographic features of the patients and healthy groups are presented in Table 1.

**Table 1:** Comparison of demographic characteristics and biochemical parameters between GH-deficient (group 1), GH-sufficient patients (group 2), and control group (group 3)

	Group 1 (*n* = *5*)	Group 2 (*n* = *25*)	Group 3 (*n* = *30*)	*P* value
Age (year)	47 ± 14.1	38.4 ± 14.1	41.3 ± 12.88	0.272*
Sex	M:5 F:0	M:21 F:4	M:25 F:5	
Body mass index (kg/m^2^)	25.0 (22.2–28.0)	26.4 (23.0–27.9)	26.0 (22.0–28.0)	0.775**
Waist circumference (cm)	90.0 (76.0–96.5)	88.0 (79.5–95.0)	88.0 (80.7–92.5)	0.979**
HOMA score	1.8 (1.3–3.9)	1.7 (0.8–2.6)	1.8 (0.8–2.2)	0.680**
Body fat ratio (%)	22.1 ± 3.5	21.0 ± 8.1	20.7 ± 9.3	0.214
Body fat mass (kg)	18.2 ± 3.0	16.0 ± 6.9	15.0 ± 8.3	0.455
Lean body mass (kg)	63.8 ± 3.9	58.9 ± 7.2	59.4 ± 9.5	0.201
Fasting blood glucose(mg/dl)	107.8 ± 36.1	90.8 ± 11.6	85.9 ± 12.5	0.150
LDL (mg/dl)	103 ± 27	103 ± 23	110 ± 26	0.573
HDL (mg/dl)	50 ± 15	49 ± 10	50 ± 13	0.902
Triglyceride (mg/dl)	93 ± 17	131 ± 40	129 ± 47	0.118
Time after trauma (months)	28.0 (26.5–45.5)	28.0 (24.5–36.0)		0.355***

Data are given as mean ± SD and median (IQR)

Group 1 = GH-deficient, group 2 = GH-sufficient patients, group 3 = control group

*M* male, *F* female, *LDL* low-density lipoprotein, *HDL* high-density lipoprotein, *HOMA* homeostatic model assessment

^*^ANOVA test is used to analyze the differences among groups. *P* value < 0.05 was considered statistically significant

^**^Kruskal–Wallis test is used to analyze the differences among groups. *P* value < 0.05 was considered statistically significant

^***^Mann–Whitney *U* test is used to analyze the differences among groups. *P* value < 0.05 was considered statistically significant

In both, the patient and control groups, basal hormone levels such as fT3, fT4, TSH, PRL, cortisol, FSH, LH, total testosterone (men), and estradiol (women) were in the normal range (Table 2). We found no TSH or FSH/LH deficiencies in patients or controls based on basal hormone levels.

**Table 2:** Comparison of basal pituitary hormones and other hormones between GH-deficient (group 1), GH-sufficient patients (group 2), and control group (group 3)

	Group 1 (*n* = *5*)	Group 2 (*n* = *25*)	Group 3 (*n* = *30*)	*P* value
TSH (mIU/ml)	1.1 (0.9–2.6)	1.0 (0.8–1.8)	1.5 (0.9–2.9)	0.206*
fT_3_ (pg/ml)	3.31 ± 0.91	3.46 ± 0.46	3.36 ± 0.37	0.562**
fT_4_ (ng/dl)	1.2 (1.0–1.3)	1.1 (1.0–1.2)	1.2 (1.0–1.3)	0.951*
IGF-1(ng/ml)	170.0 (136.5–234.0)	165.0 (139.0–218.5)	154.5 (119.7–185.0)	0.433*
Basal cortisol (µg/dl)	8.5 (7.6–16.2)	10.1 (8.5–11.2)	11.8 (9.7–13.5)	0.118*
Prolactin (ng/ml)	7.7 (7.0–15.3)	9.3 (6.4–10.9)	9.1 (8.0–13.2)	0.486*
ACTH (pg/ml)	22.6 (17.9–33.8)	18.5 (11.2–33.1)	16.6 (14.0–28.8)	0.717*
FSH (mIU/ml)	5.3 (4.6–7.7)	4.1 (2.5–10.7)	5.3 (3.0–7.9)	0.708*
LH (mIU/ml)	6.1 (4.6–6.4)	5.0 (3.8–7.2)	6.1 (4.2–7.6)	0.756*
Total testosterone^a^ (ng/dl)	575 ± 208	462 ± 144	521 ± 145	0.228**
Estradiol^b^(pg/ml)		24.3 ± 11.1	32.4 ± 16.8	0.385**
Insulin (mU/ml)	10.0 (5.6–13.0)	7.9 (4.8–12.1)	7.6 (4.5–10.2)	0.670*

Data are given as mean ± SD and median (IQR)

Group 1 = GH-deficient, group 2 = GH-sufficient patients, group 3 = control group

*ACTH *adrenocorticotropic hormone, *FSH* follicle-stimulating hormone, *GH *growth hormone, *IGF-1* insulin-like growth factor I, *LH *luteinizing hormone, *fT3* triiodothyronine, *fT4* thyroxine, *TSH* thyroid-stimulating hormone

^*^Kruskal–Wallis test is used to analyze the differences among groups. *P* value < 0.05 was considered statistically significant

^**^ANOVA test is used to compare differences among three groups. *P* value < 0.05 was considered statistically significant

^a^Total testosterone was measured only in males

^b^Estradiol levels were measured only in women at their follicular phases

**Fig. 2:**
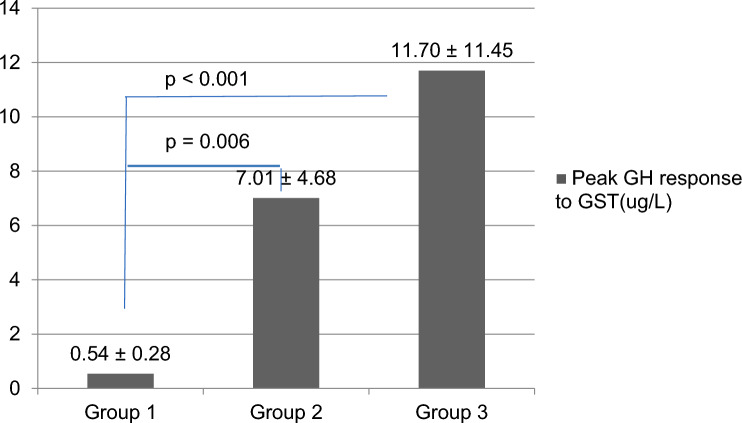
Comparison of peak GH response to glucagon stimulation test between GH-deficient (group 1), GH-sufficient patients (group 2), and control group (group 3). Mean ± SD, *GH* growth hormone, *GST* glucagon stimulation test

After the GST, 5 of 30 (16.6%) subjects had a peak GH level of less than 1.18 µg/l, indicating that they were GH deficient. Peak GH levels were significantly lower in GH-deficient (group 1) patients compared to GH-sufficient (group 2) patients (*P* = 0.006) and the control group (group 3) (*P* < 0.001). Between GH-sufficient and control groups, GH response to the GST was similar (Fig. 2).

**Fig. 3:**
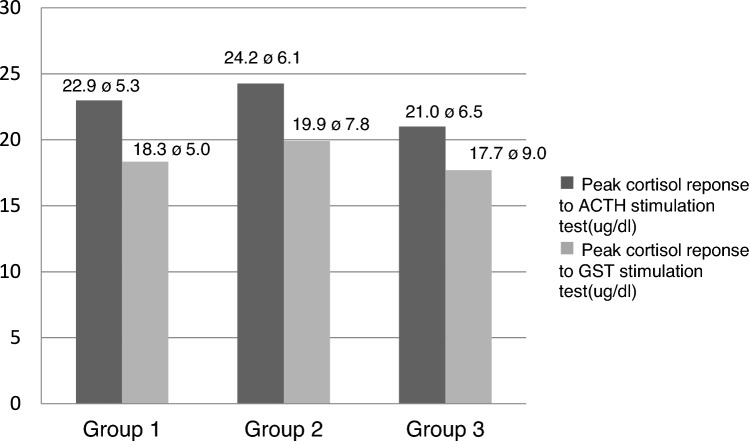
Comparison of peak cortisol response to glucagon and low-dose ACTH stimulation test between GH-deficient (group 1), GH-sufficient patients (group 2), and control group (group 3). Mean ± SD, *GST* glucagon stimulation test

All three groups’ peak cortisol responses to glucagon were not lower than the cutoff value. Furthermore, these groups were subjected to a low-dose ACTH stimulation test, and cortisol responses were not lower than the cutoff value (Table 3). These three groups’ peak cortisol responses to ACTH and glucagon stimulation tests were comparable (Fig. 3). Also, peak cortisol responses to glucagon were positively correlated with peak cortisol responses to ACTH, both in the overall sample (*r* = 0.269, *P* = 0.038) and in the patients (*r* = 0.518, *P* = 0.003). A multivariate linear regression analysis was performed to determine the effect of independent factors including age, sex, BMI, HOMA score, lipids, and basal pituitary hormones on the cortisol response to ACTH and GH response to glucagon test (*r*^2^ = 0.333 and *r*^2^ = 0.253, respectively). The analysis showed that a 1 mU/ml increase in insulin causes a 0.89 µg/dl increase in peak cortisol response (*P* = 0.035) to the ACTH test, while a 1 unit increase in HOMA score leads to a 3.22 µg/dl decrease in cortisol response (*P* = 0.025). However, no factor was found to affect GH response to the glucagon test.

**Table 3:** Peak GH response to GST, peak cortisol response to GST, and low-dose ACTH test in patients and healthy control

Patients				Control group			
No.	GH peak after GST (μg/l)	Cortisol peak after GST^b^ (μg/dl)	Cortisol peak after ACTH^c^ (μg/dl)	No.	GH peak after GST (μg/l)	Cortisol peak after GST^b^ (μg/dl)	Cortisol peak after ACTH^c^ (μg/dl)
1	3.26	14.30	15.57	1	6.2	22.4	19.4
2	2.39	19.41	23.53	2	2.1	14.0	25.0
3	2.56	20.35	29.90	3	8.1	24.0	17.8
4	10.72	22.66	35.52	4	11.0	16.0	20.0
5	2.63	15.96	20.69	5	2.1	12.8	15.0
6	6.64	40.45	34.36	6	1.6	19.0	18.0
7	5.24	17.41	19.81	7	6.5	19.0	16.0
8	4.06	37.36	29.15	8	3.4	16.0	12.0
9	15.02	27.38	37.56	9	1.9	13.0	16.0
10	5.67	19.02	27.99	10	1.8	15.0	19.0
11	11.75	11.99	19.76	11	5.4	47.0	18.0
12	***0.58***^***a***^	22.44	17.48	12	45.0	22.0	19.0
13	***0.45***^***a***^	17.39	26.37	13	9.0	17.0	24.0
14	1.45	17.88	28.01	14	36.0	26.0	12.0
15	***0.21***^***a***^	13.13	17.26	15	6.0	32.0	18.0
16	14.33	32.20	17.95	16	2.1	19.0	17.0
17	1.22	24.24	23.41	17	2.8	25.0	25.0
18	***0.5***^***a***^	14.14	24.76	18	5.6	19.0	22.0
19	***1***^***a***^	24.55	29.10	19	11.0	24.0	19.0
20	18.18	24.95	23.55	20	5.2	16.0	26.0
21	2.74	16.03	29.20	21	8.1	16.8	21.4
22	5.22	18.51	28.33	22	10.7	14.8	26.8
23	3.32	16.07	27.58	23	38.0	30.0	47.0
24	3.49	23.89	21.17	24	26.0	9.4	23.0
25	14.10	14.10	17.77	25	24.0	16.6	26.0
26	9.46	9.46	18.57	26	18.0	11.0	31.0
27	1.36	12.80	16.90	27	9.5	14.4	18.0
28	1.50	15.01	23.40	28	12.0	12.0	23.0
29	20.00	12.70	20.00	29	17.0	9.4	19.0
30	9.10	13.40	17.00	30	17.0	12.7	17.0

GS*T* glucagon stimulation test

^a^Peak GH value < 1.18 μg/l after glucagon stimulation test defined as GH deficiency

^b^Peak cortisol 9.10 μg/dl after glucagon stimulation test accepted as a cutoff value

^c^Peak cortisol 12.51 μg/dl after 1 mcg ACTH stimulation test accepted as a cutoff value

When subjects were examined individually, five (16.6%) had isolated GH deficiency. All of them were male. Two patients had zygomatic fracture, one had Le fort 1, one had a mandibular fracture, and one had a nasal fracture. Time elapsed after trauma between GH-deficient and GH-sufficient patients was similar.

Different metabolic parameters between GH-deficient and healthy subjects were investigated. Although GH-deficient patients had higher HOMA score, body mass index, body fat ratio, mean body fat mass, mean lean body mass, insulin, and fasting blood glucose levels, this was not statistically significant (Table 1).

Patients who were diagnosed with GH deficiency did not receive GH replacement treatment due to differing criteria set by national health insurance. In addition, they were not re-evaluated over time to assess if their pituitary function had returned.

## Discussion

The relationship between TBI and hypopituitarism has been known for a long time [16]. Pituitary dysfunction in patients with maxillofacial trauma has not been yet studied in literature and was observed for the first time in this study. Studies examining the relationship between TBI and pituitary functions in the literature show that 20–50% of patients have at least one pituitary hormone deficiency after TBI, with GHD being the most common [9, 11, 17]. Based on the basal hormone levels, we could not find TSH or gonadotropin deficiencies in patients with maxillofacial trauma.

The Endocrine Society clinical practice guidelines for evaluation and treatment of adult growth recommend ITT and the GHRH-arginine test to establish the diagnosis of GHD, whereas glucagon stimulation test should be limited to cases when GHRH is not available and/or performance of an ITT is either contraindicated or not practical. GST is accepted as a good alternative test for the diagnosis of GHD when GHRH is unavailable or there is a contraindication for ITT [18]. Previous literature demonstrated that GST is a reliable diagnostic test that stimulates both ACTH and GH secretion similarly to ITT [19, 20]. Also, our clinic’s recent data involving 129 patients show that the effectiveness of GST and ITT in detecting GH deficiency is similar [21]. In our study, ITT would have been impractical, especially for the control subjects. In addition, during the time of the study, GHRH-arginine was not available, which led us to use glucagon to identify GH deficiency and compare patients and control subjects.

In a retrospective study of 93 severe traumatic brain injuries, isolated hypotestosteronemia (all of which are of central origin) was found to be the most common hormone deficit, even though GH status was not assessed [22]. In a study of 15 patients with severe head trauma, the most common associated deficiencies were in gonadotropins (60%) and GH (58%) [3]. Somatotroph and gonadotroph deficiencies have been reported as the most common anterior pituitary hormone deficiencies in patients with TBI [1]. To determine GHD in kickboxers, a GST was performed, and 1.18 µg/l was found to be a cutoff value for peak GH level based on healthy subjects [11]. To avoid overdiagnosing GHD, we used our own clinic’s previously determined cutoff values. It is worth noting that cutoff values can vary depending on factors like geography and ethnicity, so using local clinic cutoffs based on healthy subjects and previous research is more reliable in our study. We discovered that five patients (16.6%) had peak GH levels of less than 1.18 µg/l in response to the GST. Peak GH responses to glucagon were above the cutoff value in the control group. Furthermore, the peak GH response to the GST in GH deficiency patients was significantly lower than the peak GH level in controls. Thus, in our study, 16.6% of the patients with maxillofacial trauma have GHD. Because GH is one of the first hormones to be lost in hypopituitarism due to a variety of causes, a lack of GH in patients with maxillofacial trauma is not surprising [23].

Hypopituitarism caused by TBI is more common than previously reported [24]. One of the primary reasons for the underestimation of TBI-induced hypopituitarism is that the clinical picture may be subtle or undetected. Indeed, hypopituitarism itself may be undiagnosed for a long time, sometimes more than 30 years [25]. It is recommended that patients with head trauma be screened for posttraumatic hypopituitarism through regular laboratory testing to confirm diagnosis [22]. In our study, glucagon and low-dose ACTH stimulation tests were performed to assess the ACTH–cortisol axis. While GST is not the first-line test for diagnosing ACTH deficiency in adults, studies have demonstrated that it can be highly sensitive (up to 98%) when the appropriate cutoff is used. In the study conducted by Karaca, Z. et al., healthy individuals displayed cortisol responses of 20.1, 12.5, and 9.1 g/dl to 250 mg ACTH, 1 mg ACTH, and glucagon stimulation tests, respectively [15]. In all groups, cortisol levels were found to be higher than the cutoff value determined in previous reports [11, 15]. Furthermore, patients with maxillofacial trauma and the control group had similar cortisol responses to stimulation tests. Therefore, we did not find ACTH deficiency in patients with maxillofacial trauma.

Multivariate linear regression analysis revealed that an increase in insulin levels leads to an increase in the peak cortisol response to the ACTH test. Contrary, an increase in the HOMA score results in a decrease in cortisol response. It is important to note that basal insulin levels can vary on a daily basis. Therefore, blood glucose and HOMA scores may be more valuable in reflecting prediabetes or insulin resistance than insulin level alone [26]. It was reported that cortisol response to stress or stimulation tests may be blunted in prediabetes which may explain the decrease in peak cortisol value with an increase in HOMA score [27, 28].

It has been shown that the metabolic effects of posttraumatic GHD occur approximately 3–5 years later [29, 30]. In adults, growth hormone deficiency is associated with abnormal body composition, decreased exercise capacity and quality of life, dyslipidemia, insulin resistance, and increased cardiovascular risk [30]. It takes some time for body composition changes or increased cardiovascular risk to occur in patients with untreated GHD. Our study found that the GH-deficient group had higher blood glucose, insulin, and HOMA scores than the healthy controls, but the difference was not significant. In these patients, GHD is early onset, and glucose, insulin, or HOMA levels could be different for other reasons prior to the trauma. In addition, multivariate linear regression analysis revealed that there were no factors affecting the GH response of the glucagon test. Neither glucose and insulin levels nor HOMA score were co-founding factors in GH deficiency. This increases the reliability of our study and shows that GH deficiency is solely due to maxillofacial trauma.

Three months after trauma, Amaretti et al. discovered normal pituitary function in 40% of mild, moderate, and severe TBI patients. After 12 months, 60% of the patients had normal pituitary function. In contrast, hormone deficiency was found in 5.5% of patients with normal pituitary function 3 months after TBI at 12 months [5]. Tanrıverdi et al. assessed pituitary functions in the first and fifth years of patients with TBI. It was observed that some of the patients who had hormone deficiency in the first year had normal levels in the fifth year. However, in patients who had no deficiency in the first year, the deficiency appeared in the fifth year [29]. TBI may cause temporary or permanent anterior and/or posterior pituitary insufficiency [16]. In our study, patients were assessed after an average of 2 years from trauma. Iglesias et al. reported a patient who recovered spontaneously from anterior pituitary hormone secretion was achieved 6 months after head trauma. In this patient, insufficient GH responses to the pituitary and hypothalamic stimuli suggest temporary hypothalamic and pituitary damage [8]. Thus, patients with maxillofacial head trauma should be monitored for hypopituitarism for 5 years after the incident.

For the first time in the literature, GHD was discovered in patients with maxillofacial trauma in this study. This result was similar to pituitary insufficiency in patients with head trauma for various reasons in the literature. However, more patients and prospective studies are required to understand the natural course and frequency of pituitary insufficiency following maxillofacial head injuries. Also, further studies are needed to establish the diagnosis of GHD with ITT and/or the GHRH-arginine test in patients with maxillofacial fractures.

The most significant limitation of this study is the insufficient number of patients because it was difficult to find a patient with a maxillofacial fracture and mild head trauma. The fact that the patients were selected from a certain geographical region impairs the generalizability of our results.

In conclusion, physicians should be on the lookout for pituitary insufficiency in patients with maxillofacial trauma, particularly GHD. Therefore, these patients should be evaluated for pituitary dysfunction after trauma.

## Data Availability

Some or all datasets generated during and/or analyzed during the current study are not publicly available but are available from the corresponding author upon reasonable request.
